# Tuberculosis Trends in Saudis and Non-Saudis in the Kingdom of Saudi Arabia – A 10 Year Retrospective Study (2000–2009)

**DOI:** 10.1371/journal.pone.0039478

**Published:** 2012-06-20

**Authors:** Mohammad S. Abouzeid, Alimuddin I. Zumla, Shaza Felemban, Badriah Alotaibi, Justin O’Grady, Ziad A. Memish

**Affiliations:** 1 Preventive Medicine Directorate, Ministry of Health, Riyadh, Saudi Arabia; 2 Division of Infection and Immunity, Department of Infection, University College London, London, United Kingdom; 3 College of Medicine, Alfaisal University, Riyadh, Saudi Arabia; Public Health Agency of Barcelona, Spain

## Abstract

**Background:**

Tuberculosis (TB) remains a public health problem in the Kingdom of Saudi Arabia (KSA), which has a very large labour force from high TB endemic countries. Understanding the epidemiological and clinical features of the TB problem, and the TB burden in the immigrant workforce, is necessary for improved planning and implementation of TB services and prevention measures.

**Methods:**

A 10 year retrospective study of all TB cases reported in KSA covering the period 1st January 2000 to 31st December 2009. Data was obtained from TB reporting forms returned to the Ministry of Health. Data were then organised, tabulated and analysed for annual incidence rates by province, nationality, country of origin and gender.

**Results:**

There was an annual increase in the number of TB cases registered from 3,284 in 2000 to 3,964 in 2009. Non-Saudis had nearly twice the TB incidence rate compared to Saudis (P = <0.05). All but four provinces (Najran, Riyadh, Makkah, Tabuk) showed decreasing TB incidence rates. The highest rates were seen in the 65+ age group. In the 15–24 year age group the incidence rate increased from 15.7/100,000 in 2000 to 20.9/100,00 in 2009 (P = <0.05). The incidence of TB in Saudi males was higher than Saudi females. Conversely, for non-Saudis the TB incidence rates were significantly higher in females compared to males.

**Conclusions:**

Despite significant investments in TB control over 15 years, TB remains an important public health problem in the KSA affecting all age groups, and Saudis and non-Saudis alike. Identification of the major risk factors associated with the persistently high TB rates in workers migrating to KSA is required. Further studies are warranted to delineate whether such patients re-activate latent *Mycobacterium tuberculosis (M.tb)* infection or acquire new *M.tb* infection after arrival in KSA. Appropriate interventions are required to reduce TB incidence rates as have been implemented by other countries.

## Introduction

Despite the declaration of tuberculosis (TB) as a global emergency by the World Health Organisation (WHO) in 1993 [1], TB caused 1.45 million deaths in 2010 and remains a major international health problem. Globally, the average incidence rate of TB peaked at 142 cases per 100,000 people in 2004. Since then, it has fallen by approximately 1 per cent a year, reaching 137 cases per 100,000 people in 2009 [2]. There were an estimated 9.4 million incident cases of TB globally in 2009, most of which were in the South-East Asia, African and Western Pacific regions (35%, 30% and 20%, respectively). There were 5.8 million cases notified in 2009, of which 2.6 million cases were new pulmonary sputum smear-positive, 2.0 million were new pulmonary sputum smear-negative, 0.9 million were new extra-pulmonary (EPTB) and 0.3 million were relapse cases. Most new cases were reported in Asia (55%) and Africa (30%). Global targets for reducing the TB burden, as set by the Millennium Development Goals (MDGs), are to halt and reverse the incidence by 2015. Additional targets have been set by the Stop TB Partnership. These targets are to halve the prevalence and death rates by 2015, compared with their levels in 1990, and to reduce the global incidence of active TB to one case per one million populations by the year 2050 [2].

The five countries with the largest numbers of TB cases are India, China, South Africa, Nigeria and Indonesia. China and India combined account for approximately 35% of the world’s new TB cases [3]. The Kingdom of Saudi Arabia (KSA) is the biggest employer of a huge migrant labour force of all grades of workers from several high TB endemic Asian, South-East Asian, African and Middle eastern countries. The total population of KSA was 20,378,956 in 2000 (of which 5,548,086 were non-Saudis), and was 25,373,512 in 2009 (of which 8,589,817 were non Saudis). The KSA is a large country constituting the main bulk of the Arabian Peninsula. It is divided into thirteen provinces the main populous ones being Riyadh, Makkah, Al-Madinah, Aseer, Al-Baha, Ha'il, Al-Jouf, Jizan, Najran, Qaseem, Tabouk in addition to Eastern and Northern provinces. Each province has one General Directorate for Health Affairs and considered as one notification unit.

KSA has applied a National TB Control Program (NTP), the activities of which are integrated in the general health care facilities. The program is directed and supervised by a central unit located in the Preventive Medicine Directorate, Ministry of Health. The tasks of the central unit include; policy making, monitoring, evaluation, training, and coordination with the different levels of the governmental health system and other sectors that may be involved in TB control activities [4]. Due to the social stigma accompanying TB in KSA, TB care services are integrated in general health care facilities. There are only two chest hospitals (in Riyadh and Taif) managing patients with chest diseases, including TB. These two hospitals are considered referral hospitals for managing MDR-TB nationwide. There are also two TB centres (in Jeddah and Al-Hassa), considered as outpatient clinics. Identification of TB suspects is performed at all levels of health care service levels by general practitioners and specialities. Diagnosis of TB patients and prescription of treatment is only performed in hospitals by specialists, while follow-up is undertaken in hospitals and by general practitioners in primary health care centres. Diagnosis of TB is based mainly on sputum smear microscopy, radiography, mycobacterial culture, histopathology, in addition to molecular techniques available in some referral laboratories. Surveillance starts at the outpatient clinics and in primary health care centres by general practitioners. Patients suspected of TB are listed in a suspect register and referred to the nearby hospitals for diagnosis. Confirmed TB patients are notified to an assigned co-ordinator in the same hospital. If there is no assigned co-ordinator, diagnosed cases are notified to the infection control section, who notify to the district co-ordinator, using the adopted notification form. After notification, the district co-ordinator assigns the case a unique number in the TB register and informs the hospital co-coordinator of that number in order to label the patient’s treatment card. Patients are given appointments by the treatment physicians for follow-up and smear examination. Smear microscopy results are kept in the patient file and a copy is sent to the district co-ordinator to be recorded in the TB register. District co-ordinator submits a monthly case-based report to the central unit including new and relapse patients in addition to treatment outcome. Patients who default on treatment are traced and contacted by an outpatient clinic nurse and the district TB co-ordinator.

Whilst TB continues to be a public health problem in KSA, published studies on TB in Saudi Arabia [5]–[10] have concentrated on extra-pulmonary TB (EPTB) and drug susceptibility testing. No data has been published to date on TB in the large migrant labour force, or its impact on TB incidence trends in KSA among Saudi nationals. Understanding the epidemiological and clinical features of the TB problem, and the TB burden in the immigrant workforce, is necessary for improved planning and implementation of TB services and prevention measures. In this study, a 10 year retrospective analysis of all TB cases reported in KSA to assess trends in incidence rates by province, nationality, country of origin and gender.

## Methods

A retrospective 10 year study of all TB cases reported in KSA during the period 1^st^ January 2000 to 31^st^ December 2009. The study included only new TB cases, recurrent cases and relapse were not included. KSA has mandatory reporting of all TB cases and returns are made monthly and notified by name to the central unit of the programme and then entered into an Epi-info programme. Patients are classified according to WHO criteria using a patient code, microbiological results and X-ray results. TB cases are notified to the NTP once the clinician makes the decision to treat the patient for TB. Data were obtained from TB reporting forms returned to the Ministry of Health. Data were then organised, tabulated and analysed for annual incidence rates by province, nationality, country of origin and gender. Data were then organized, tabulated and aggregated using SPSS statistical package, version 16. Definitions adopted by WHO for TB cases classification [3] and the incidence rates per 100,000 populations were calculated. Chi Square was used for linear trends, Pearson's Chi Square technique and Poisson log linear regression were used as appropriate. The significance level was set at 0.05.

### Denominators used to calculate the rates of period

Saudi Arabia carried out three censuses in 1413H (1992), 1425H (2004) and 1431H (2010). In between censuses, the Central Department of Statistics & Information estimates the population. Population statistics were obtained from the Central Department of Statistics & Information, Ministry of Economy & Planning, Saudi Arabia. The population data included the number of population by province, nationality (national or non-national), age and sex for the period of the study. Population data for non-Saudis were provided collectively, without details about the country of origin, by province of residence in the Kingdom, age and sex. These data were used as denominators to calculate the incidence rates in the study.

### Ethics statement

Ethical approval or written patient consent were not required for the study as this was a retrospective review of routine surveillance data collected anonymously by the KSA NTP. The KSA Ministry of Health has a mandate to review and evaluate the NTP.

## Results

The total number of TB cases and TB incidence rates per 100,000 population by year reported in Saudis and non-Saudis resident in KSA are presented in **Table 1**. There was a yearly increase in the number of TB cases registered with an increase in numbers from 3,284 in 2000 to 3,964 in 2009. Although not statistically significant, the TB incidence rates showed a gradual decline for the years 2000–2004, dropping to 14.2/100,000 in 2004 followed by a gradual rise from 2005 reaching a peak of 16/100,000 in 2008. Non-Saudis had nearly twice the incidence rate compared to Saudis (P = <0.05), throughout the study period, although the rates in either group did not significantly increase (**Table 1**).

**Table 1:** Total number of TB and incidence rate/100,000 in Saudi Arabia (2000–2009) by nationality.

Year	Saudi				Non-Saudi				Total		Non-Saudi/Total (%)
	No.	% change	rate	% change	No.	% change	rate	% change	No.	rate	
**2000**	1729	-	11.7	-	1555	-	28	-	3284	16.1	47.4%
**2001**	1734	0.30%	11.4	−2.60%	1487	−4.40%	26.1	−6.80%	3221	15.4	46.2%
**2002**	1726	−0.50%	11.1	−2.60%	1540	3.60%	26.4	1.10%	3266	15.2	47.2%
**2003**	1747	1.20%	10.9	−1.80%	1447	−6.00%	24.2	−8.30%	3194	14.5	45.3%
**2004**	1720	−1.50%	10.5	−3.70%	1487	2.80%	24.3	0.40%	3207	14.2	46.4%
**2005**	1873	8.90%	11.1	5.70%	1587	6.70%	25.3	4.10%	3460	15	45.9%
**2006**	1989	6.20%	11.5	3.60%	1648	3.80%	25.7	1.60%	3637	15.4	45.3%
**2007**	2049	3.00%	11.6	0.90%	1798	9.10%	27.4	6.60%	3847	15.9	46.7%
**2008**	2155	5.20%	11.9	2.60%	1802	0.20%	26.9	−1.80%	3957	16	45.5%
**2009**	2137	−0.80%	11.5	−3.40%	1827	1.40%	26.7	−0.70%	3964	15.6	46.1%
κ^2^*			3.1				0.5		2.7		6.9
P			>0.05				>0.05		>0.05		>0.05

* Chi-Square for linear trend.

**Table 2** depicts the cumulative proportion of TB cases in non-Saudis, resident in KSA, by country of origin. Non-Saudi TB cases originated from Indonesia (16.68%), Yemen (11.14%), India (12%), Pakistan (9.21%), Bangladesh (8.1%), Philippines (6.84%), Ethiopia (5.42%), Somalia (5.17%), Chad (4.96%), Sudan (4.73%), Nigeria (3.81%) and other African countries (13.46%).

**Table 2:** Cumulative proportion of non-Saudi TB patients by country of origin (2000–2009).

Country of origin	Number (%) of TB
Indonesia	3045 (16.68%)
Yemen	2033 (11.14%)
India	1915 (10.49%)
Pakistan	1682 (9.21%)
Bangladesh	1474 (8.10%)
Philippines	1249 (6.84%)
Ethiopia	991 (5.42%)
Somalia	944 (5.17%
Chad	906 (4.96%)
Sudan	864 (4.73%)
Nigeria	696 (3.81%)
Other African countries	2,457 (13.46%)
Total (18,256)	

**Figure 1:**
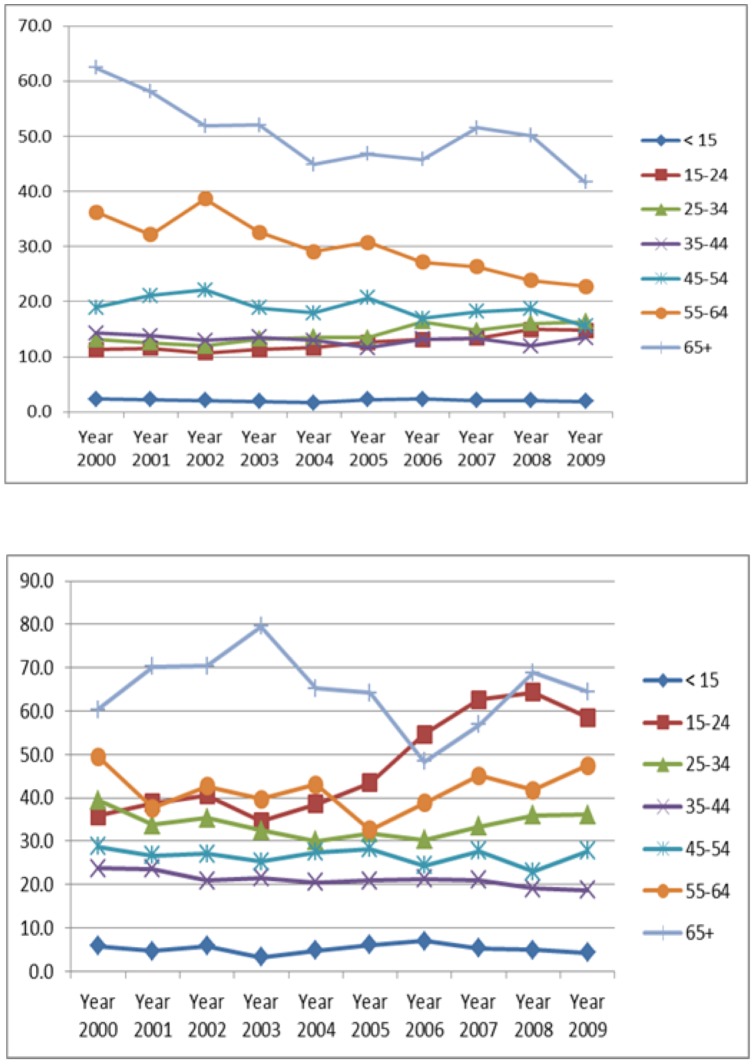
(A) Annual TB incidence rate trends among Saudis (2000–2009); (B) Annual TB incidence rate trends among non-Saudis (2000–2009).

The total number of TB cases and TB incidence rates were broken down by age groups (**Table 3**). The highest rates were observed in the 65+ age group at 65.2/100,000 in 2000. In the 15–24 year age group, the incidence rate increased from 15.7/100,000 in 2000 to 20.9/100,00 in 2009 (P = <0.05). In all other age groups the incident rates showed declining rates. The most significant decline (P = <0.05) was in the 65+ age group where rates fell from 65.2/100,000 to 43.9/100,000 in 2009. The number of cases by age distribution between Saudis and non-Saudis is shown in Figures 1a and 1b. The overall incidence rates among both Saudis and non-Saudi shows no significant changes over the study period. Among Saudis, the rate was found to increase with the progression of age with a significant declining trend over years, except for age group below 15 years which shows no significant changes over the study period. In contrast, among non-Saudis the incidence rate was found to decrease with the progression of age with no significant changes over years, except for age group 15–24 years which shows a rising trend and age group 35–44 years which shows a declining trend.

**Table 3:** Total number and incidence rate of TB cases in Saudi Arabia (2000–2009) by year and age group.

Year	Age group													
	<15		15–24		25–34		35–44		45–54		55–64		65+	
	n	rate	n	rate	n	rate	n	rate	n	rate	n	rate	n	rate
**2000**	196	2.9	598	15.7	963	24.1	546	18.6	352	22.6	276	39	353	65.2
**2001**	186	2.7	645	16.5	880	21.5	552	18.3	371	23.2	242	33.3	345	59.2
**2002**	191	2.8	644	16.1	913	21.7	512	16.6	392	24	294	39.4	320	53.6
**2003**	154	2.1	634	15.4	914	21.2	542	17.2	356	21.2	260	34	334	54.5
**2004**	162	2.1	693	16.4	903	20.4	533	16.5	371	21.5	251	32	294	46.7
**2005**	221	2.9	751	17.7	958	21.1	538	15.9	425	23.5	256	31.2	311	48.4
**2006**	238	3.1	841	19.5	1026	22	599	17	374	19.7	256	29.7	303	46
**2007**	208	2.6	902	20.7	1052	22.2	623	16.9	434	21.8	277	30.5	351	52
**2008**	202	2.5	978	22.1	1137	23.8	588	15.3	424	20.3	268	28	360	51.9
**2009**	189	2.3	941	20.9	1148	23.8	641	16	441	20.1	291	28.7	313	43.9
**% change**	3.6	20.7	57.4	33.1	19.2	1.2	17.4	14.0	25.3	11.1	5.4	26.4	11.3	32.7
χ^2^*	0.5		108		2.9		12		8.3		20.9		21	
P	>0.05		<0.05		>0.05		<0.05		<0.05		<0.05		<0.05	

* Chi-Square for linear trend.

The incidence of TB in Saudi males was higher than Saudi females. Conversely, for non-Saudis, the TB incidence rates were significantly higher in females compared to males (**Table 4**).

**Table 4:** Total number and incidence rate/100.000 of TB cases in Saudi Arabia (2000–2009) by nationality and sex.

Year	Saudi				non-Saudi				Male/female Sex ratio
	male		female		male		female		
	No.	rate	No.	rate	No.	rate	No.	rate	
**2000**	899	12.1	830	11.2	872	22.6	683	40.4	1.2
**2001**	940	12.4	794	10.4	835	21.2	652	37.3	1.2
**2002**	922	11.8	804	10.3	921	22.9	619	34.4	1.3
**2003**	936	11.7	811	10.1	845	20.5	602	32.4	1.3
**2004**	958	11.6	762	9.3	840	19.8	647	34.6	1.3
**2005**	1043	12.3	830	9.9	876	20.2	711	36.9	1.3
**2006**	1178	13.6	811	9.4	884	20.0	764	38.6	1.3
**2007**	1169	13.3	880	9.9	969	21.5	829	40.7	1.3
**2008**	1219	13.4	936	10.4	1001	21.8	801	38.3	1.3
**2009**	1241	13.3	896	9.7	1077	23.0	750	35.0	1.4
**χ^2^ for linear trend**	23.1		6.6		0.02		0.13		
**P**	<0.05		<0.05		>0.05		>0.05		

**Table 5** shows the number of cases of smear positive TB, smear negative TB and extrapulmonary TB (EPTB) by year, in Saudis and non-Saudis. The number of cases and proportions of pulmonary sputum smear positive TB cases increased among both Saudis and non-Saudis over the 10 year period. The numbers of EPTB cases showed an upward trend among Saudis (9% change) compared to a downward trend among non-Saudis (17.2% change).

**Table 5:** Number and percentage of TB cases in Saudi Arabia (2000–2010) by form of TB and nationality.

Year	Saudi						non-Saudi					
	SS+		SS−		EPTB		SS+		SS−		EPTB	
	No.	%	No.	%	No.	%	No.	%	No.	%	No.	%
**2000**	777	44.9%	434	25.1%	518	30.0%	810	52.1%	264	17.0%	481	30.9%
**2001**	816	47.1%	370	21.3%	548	31.6%	872	58.6%	212	14.3%	403	27.1%
**2002**	801	46.4%	387	22.4%	538	31.2%	877	56.9%	201	13.1%	462	30.0%
**2003**	850	48.7%	359	20.5%	538	30.8%	802	55.4%	183	12.6%	462	31.9%
**2004**	828	48.1%	348	20.2%	544	31.6%	862	58.0%	235	15.8%	390	26.2%
**2005**	891	47.6%	374	20.0%	608	32.5%	845	53.2%	274	17.3%	468	29.5%
**2006**	971	48.8%	387	19.5%	631	31.7%	934	56.7%	273	16.6%	441	26.8%
**2007**	988	48.2%	362	17.7%	699	34.1%	1004	55.8%	218	12.1%	576	32.0%
**2008**	1061	49.2%	358	16.6%	736	34.2%	1068	59.3%	190	10.5%	544	30.2%
**2009**	1072	50.2%	366	17.1%	699	32.7%	1143	62.6%	216	11.8%	468	25.6%
**% change**	38%	11.8%	-15.7%	- 31.9%	34.9%	9%	41.1%	20.2%	-18.2%	-30.6%	-2.7%	-17.2%
**χ^2^_18_** *	74.5						115.6					
**P**	<0.05						<0.05					

* Pearson Chi-square.

The total number and incidence rate per 100,000 population was analysed by province (**Table 6**). Makkah and Riyadh provinces constitute nearly 70% of all TB cases reported. The highest incidence rates throughout the study period were in: Makkah (range 22–25.2/100.000), Jizan (range 18.4–18.7/1000.000), Riyadh (15.7 to 17.8/100.000), Northern Province (18.4 to 12.1/100.000), Qaseem (range 16.6 to 11.3/100.000) and Tabuk (range 14.9 to 11.2/100.000). The incidence rates increased significantly in Najran, Makkah, Riyadh, and Tabuk. All other provinces showed a declining trends with the greatest decline in Ha'il (from 14.7/100,000 in 2000 to 4/100,000 in 2009) followed by Al-Jouf, Al-Baha and Northern provinces.

**Table 6:** Total number and incidence rate of TB cases in Saudi Arabia (2000–2009) by Province.

Province		Year										% change 00–09	κ^2^ *	
		2000	2001	2002	2003	2004	2005	206	2007	2008	2009			P
**Riyadh**	n	771	790	839	747	705	898	883	935	1035	1110	44.0%	13.6	<0.05
	Rate	15.7	15.7	16.2	14.1	13	16	15.3	15.8	17	17.8	13.4%		
**Makkah**	n	1150	1035	1096	1141	1325	1262	1544	1738	1665	1599	39.0%	143.7	<0.05
	Rate	22	19.3	20	20.3	22.9	21.5	25.8	28.4	26.7	25.2	14.5%		
**Al-Madinah**	n	181	228	165	134	139	190	216	156	164	175	−3.3%	17.9	<0.05
	Rate	13.4	16.4	11.5	9.1	9.2	12.3	13.6	9.6	9.8	10.2	−23.9%		
**Qaseem**	n	151	139	136	150	108	122	136	124	130	128	−15.2%	18.5	<0.05
	Rate	16.6	14.9	14.2	15.2	10.7	11.8	12.8	11.4	11.7	11.3	−31.9%		
**Eastern**	n	396	397	330	315	330	326	296	315	332	334	−15.7%	52.5	<0.05
	Rate	13.1	12.8	10.4	9.7	9.9	9.5	8.5	8.8	9.1	9	−31.3%		
**Aseer**	n	120	146	170	149	121	142	105	112	145	132	10.0%	14.8	<0.05
	Rate	8	9.4	10.7	9.1	7.2	8.3	6	6.3	8	7.1	−11.3%		
**Tabuk**	n	92	111	127	112	88	88	84	96	107	89	−3.3%	13.7	<0.05
	Rate	14.9	17.5	19.5	16.7	12.8	12.4	11.5	12.8	13.8	11.2	−24.8%		
**Ha'il**	n	69	63	56	58	53	53	61	40	38	23	−66.7%	43.8	<0.05
	Rate	14.7	13	11.3	11.4	10.1	9.9	10.9	7.2	6.7	4	−72.8%		
**Northern**	n	46	39	42	36	41	38	41	51	43	37	−19.6%	1.7	>0.05
	Rate	18.4	15.2	15.9	13.3	14.7	13.4	14.2	17.3	14.3	12.1	−34.2%		
**Jazan**	n	195	171	192	222	191	212	187	193	200	255	30.8%	1.1	>0.05
	Rate	18.4	15.7	17.1	19.3	16.2	17.4	14.9	15	15.1	18.7	1.6%		
**Najran**	n	17	16	14	37	28	24	30	21	34	39	129.4%	5.1	<0.05
	Rate	4.5	4.1	3.5	9.1	6.7	5.6	6.7	4.6	7.2	8	77.8%		
**Al-Baha**	n	36	41	41	43	33	62	28	32	33	18	−50.0%	13.2	<0.05
	Rate	10.7	11.8	11.5	11.7	8.8	16.2	7.2	8.1	8.3	4.4	−58.9%		
**Al-Joof**	n	60	45	58	50	45	43	26	34	31	25	−58.3%	31.2	<0.05
	Rate	18.6	13.5	17	14.3	12.5	11.6	6.9	8.7	7.8	6.1	−67.2%		

* Chi square for trend.

## Discussion

Our study revealed five significant findings: 1) TB remains an important public health problem in the KSA, affecting all age groups, and Saudis and non-Saudis alike; 2) non-Saudis had nearly twice the TB incidence rate compared to Saudis throughout the study period; 3) non-Saudi TB patients originated from high TB endemic areas of the world, particularly Indonesia, India, Pakistan, Bangladesh, Philippines, Ethiopia, Somalia, Chad, Nigeria and other African countries; 4) the incidence of TB in Saudi males was higher than Saudi females and, conversely, the TB incidence rates in non-Saudis were significantly higher in females compared to males and; 5) Makkah and Riyadh provinces, cities attracting a large proportion of migrant workers, constitute nearly 70% of all TB cases reported from the Kingdom and TB incidence rates have increased over the past decade in these provinces.

This is the first detailed analyses of official TB surveillance data from KSA for the period 2000–2009. There have been no previous reports of TB trends in KSA or the importance of TB within the large migrant labour force, and its contributions to, and impact on TB incidence trends in KSA among Saudi nationals. Understanding the epidemiological and clinical features of the TB problem, and the TB burden in the immigrant workforce, is necessary for improved planning and implementation of TB services and prevention measures. The varying TB rates seen in different geographical areas of the KSA, the differences in rates between Saudis and non-Saudis and the gender differences may due to several reason including the quality of the NTP diagnostic and health services in these areas, access to health services, social and housing factors and population density. Important confounding risk factors for TB are poverty, poor living conditions and income level and further studies on evaluating common social determinants of TB could help explain some of the differences between TB rates in Saudis and non-Saudis. Stress and poor nutrition may lead to reactivation of latent *M.tb* infection (LTBI) and improvements in living conditions and income levels of the migrant workforce combined with screening and preventive treatment for LTBI could help control TB in the non-Saudi population. Illegal residents and migrants in any country live in poor conditions and do not regularly access health care for fear of being deported and the health problems of this population is ongoing. The KSA NTP is currently evaluating the burden of TB and HIV and the knowledge, attitudes and practices amongst illegal residents in major KSA cities. This information will guide more investments into improving health care for undocumented migrants [11]

The KSA NTP was established in the early nineteen seventies [4]. Directly observed treatment, short course (DOTS) was fully adopted and implemented in a number of demonstration provinces in 1998 [11] and expanded throughout KSA by the beginning of the year 2000 [12]. Despite appropriate investments into a functional and effective TB service, the incidence of TB in KSA is not declining. This suggests a more comprehensive assessment of the NTP in KSA is required to ascertain the programmatic and operational issues that have hampered the decline of TB rates over the past decade.

Before 2000, non-Saudis were not allowed to reside permanently in the Kingdom [13]. They were not allowed access to free government healthcare facilities, and they did not access private healthcare facilities for fear of deportation. Consequently, many non-Saudi TB patients were not diagnosed or registered, leading to under reporting. Since the end of 2000, non-Saudi patients with symptoms suggestive of TB were allowed to access governmental healthcare facilities free of charge and were allowed to remain in the Kingdom [14]–[15]. This policy change encouraged non-Saudis to use government health care facilities, thus increasing case detection rates in non-Saudis. TB education was strengthened in KSA in order to alleviate the social stigma around the disease, educate people about symptoms and encourage more Saudis and non-Saudis to be tested for TB.

The strengthening of the laboratory network and intensification of the surveillance system and health education activities has led to an increase in the number of the TB cases registered, however increases in population resulted in a stable incidence rate during the study period. In countries where effective TB control measures are applied, such as Beijing [16], Cuba [17] and Peru [18], a rapid decline in TB incidence have been reported.

Non-Saudis have higher TB incidence rates than Saudis. This is most likely related to the high TB incidence rate in the country of origin [19], as many of the non-Saudis may have LTBI and develop active TB while residing in KSA for reasons such as: stress, poor housing conditions, poor nutrition, or immunosuppressive treatments [20]–[21]. A proportion may also represent TB caused by newly acquired *M.tb* infection due to the high TB incidence rates in major KSA cities. As the overall incidence rate among both Saudis and non-Saudis is stable, the incidence rate among those aged below 15 years is very low and the highest incidence rate reported among elderly, it is assumed that reactivation of latent *M.tb* infections may be important. Further genotyping studies clustering secondary cases to index cases will clearly delineate this issue. Molecular studies on *M.tb* isolates will establish whether the main issue is re-infection or re-activation. Re-activation of LTBI could be prevented by screening for latency at points of entry and administering preventive therapy. Screening of migrant non-Saudis for active TB at six monthly intervals will enhance case detection rates, allowing early treatment and reduction of transmission.

The observed gender differences are more difficult to explain. The low total numbers and incidence rates for Saudi females may reflect their reduced participation in KSA public and society. It may also reflect the stigma associated with being diagnosed with TB as a Saudi female. In contrast, non-Saudi females have higher incidence than males, a finding that may be linked to lower socioeconomic status relating to occupation – most of the affected non-Saudi females were servants or jobless (44% and 21%, respectively). Additionally, most of non-Saudi females originated from countries with high burden of TB. Among Saudis, males had higher TB rates than females, indicating a consequence of the societal role of both sexes [22]. Males generally are known to be more susceptible to TB than females because of their relatively large social network that increases their risk of infection [23], in addition to the higher prevalence of smoking, which has a confirmed association with TB [24]–[26].

The proportion of pulmonary smear positive TB among Saudis is consistent with that reported globally (45.5%) but higher than that reported in the Eastern Mediterranean region (38.1%) [3]. On the other hand, the proportion of pulmonary smear negative TB (16.6%−25.1%) was lower than that reported both globally and regionally (34.7% and 40.3%, respectively). Regarding EPTB, the proportion reported among Saudis (30% −34.2%) was higher than that reported globally and regionally (14.8% and 18.9%, respectively). Among non-Saudis, the proportion of pulmonary smear positive TB and EPTB were higher than that reported globally and regionally while that of pulmonary smear negative TB was lower. These observations are difficult to explain and may reflect inconsistencies in clinical practice and quality of diagnostic services.

The proportion of pulmonary smear positive TB shows an upward trend among both Saudis and non-Saudis (11.8% and 20.2% change, respectively), indicating the effect of intensifying the microscopy laboratory network and services. On the other hand, the proportions of pulmonary smear negative and extrapulmonary forms show a downward trend among both Saudis and non-Saudis. The decline in proportion of pulmonary smear negative and extrapulmonary forms was higher among non-Saudis compared to Saudis. Difficulties facing non-Saudis in accessing health care facilities may attribute to this difference. The significant increasing proportion of smear positive TB could be attributed to strengthening of the microscopy laboratory network in the kingdom through adding new peripheral laboratories, providing liquid mycobacterial culture equipment (MGIT), intensifying on job training for technicians and implementation of a quality control program in laboratories. Becoming symptomatic early in the course of the disease, smear positive pulmonary tuberculosis is usually diagnosed early as the procedures for its diagnosis is straight forward, regardless of the nationality and healthcare facility. In contrast, smear negative pulmonary and extrapulmonary TB requires more complicated diagnostic procedures, including hospitalization, which may lead to delayed diagnosis.

Makkah and Riyadh provinces, the largest regions with the biggest populations in KSA, attract a large workforce including nationals and non-nationals resulting in increased *M.tb* transmission rates. Furthermore the medical and laboratory services are better with staff more aware of TB which facilitates increased case detection rates. As Makkah is visited by millions of pilgrims each year for the Hajj and Umrah festivals, one could attribute a proportion of the high TB rates to *M.tb* transmission to local residents during these festivals [27]. This requires further study although the religious festivals may not be the only reason for the high TB rates. Pilgrims have free access to the government health care facilities and TB is a notifiable disease, even among pilgrims. Al-Madinah is also a Hajj area but the TB incidence rate and number of notified TB cases is not above average. The frontier provinces, are less attractive to migrant workers which may explain the low TB numbers and rates.

Our study describes the current situation and trend of TB in the Kingdom of Saudi Arabia. As the incidence rate shows a stable overall trend over the years of the study it is supposed that interventions applied by NTP are not sufficient. Further studies to comprehensively review the NTP in the Kingdom are recommended to identify areas of weakness and policies that need to be changed. Also, studies to define risk factors, delay in diagnosis, and annual risk of new *M.tb* infection or reactivation of LTBI are required in addition to implementation of a quality assurance programme at all levels of TB care, including that for disadvantaged poor populations. Although, all non-Saudis seeking residency in the Kingdom have to be screened for TB by chest radiography in their home country, the efficacy of this programme needs to be evaluated, and more effective preventive measures applied.

### Conclusion

Despite significant investments in TB control over 15 years, TB remains an important public health problem in KSA affecting all age groups, and Saudis and non-Saudis alike. Identification of the major risk factors associated with the persistently high TB rates in workers migrating to KSA is required. The first step in achieving this is regular screening of migrants for active and latent TB after arrival in the Kingdom. Further studies can then be performed to delineate whether these migrants re-activate latent TB or acquire the disease after arrival in KSA. A thorough assessment of the National TB Programme is required to identify weaknesses in staff knowledge and quality of clinical and laboratory services and reporting systems. Appropriate interventions are required to reduce TB incidence rates as have been implemented by other countries.
